# The nucleotide exchange factors of Hsp70 molecular chaperones

**DOI:** 10.3389/fmolb.2015.00010

**Published:** 2015-04-07

**Authors:** Andreas Bracher, Jacob Verghese

**Affiliations:** Department of Cellular Biochemistry, Max-Planck-Institute of BiochemistryMartinsried, Germany

**Keywords:** BAG domain, cochaperone, GrpE, HspBP1/Sil1, Hsp110/Grp170, protein folding, proteostasis

## Abstract

Molecular chaperones of the Hsp70 family form an important hub in the cellular protein folding networks in bacteria and eukaryotes, connecting translation with the downstream machineries of protein folding and degradation. The Hsp70 folding cycle is driven by two types of cochaperones: J-domain proteins stimulate ATP hydrolysis by Hsp70, while nucleotide exchange factors (NEFs) promote replacement of Hsp70-bound ADP with ATP. Bacteria and organelles of bacterial origin have only one known NEF type for Hsp70, GrpE. In contrast, a large diversity of Hsp70 NEFs has been discovered in the eukaryotic cell. These NEFs belong to the Hsp110/Grp170, HspBP1/Sil1, and BAG domain protein families. In this short review we compare the structures and molecular mechanisms of nucleotide exchange factors for Hsp70 and discuss how these cochaperones contribute to protein folding and quality control in the cell.

## Introduction

**Figure 1:**
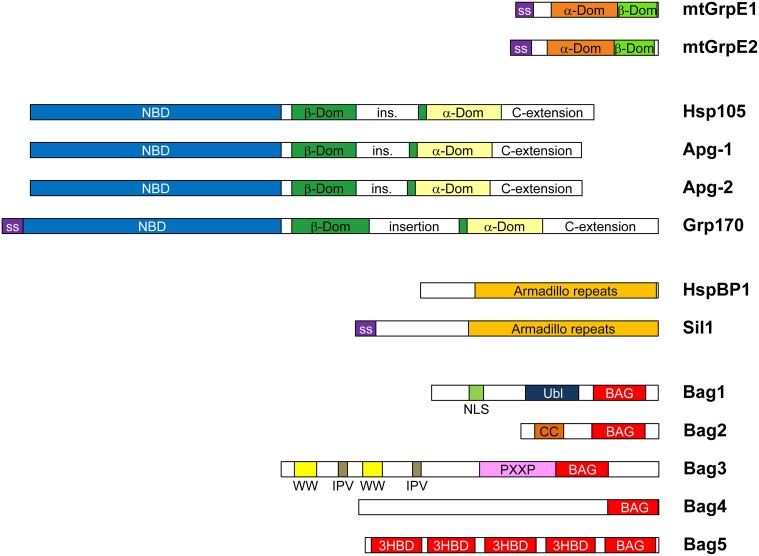
**Domain compositions of human NEF homologs**. Human cells express two mitochondrial GrpE isoforms (mtGrpE1/GRPEL1 and mtGrpE2/GRPEL2), three Hsp110 homologs (Hsp105/HSPH1, Apg-1/HSPA4L, and Apg-2/HSPA4—the codes designate the gene names) and five BAG-domain proteins. There is only one form of Grp170/HYOU1, HspBP1, and Sil1, respectively. Isoforms arising from alternative initiation sites and splicing were described for Hsp105, HspBP1, and Bag1 (not shown). mtGrpE isoforms contain mitochondrial signal sequences (ss). The α- and β-domains (orange and green, respectively) are conserved with GrpE from *E. coli*. The Hsp110/Grp170 family proteins consist of an N-terminal nucleotide binding domain (NBD, blue), a β-sandwich (β-Dom, green) and a α-helix bundle domain (α-Dom, pale yellow). All isoforms contain long variable insertions in the β-sandwich and at the C-terminus. SS indicates signal sequences for ER import of Grp170 and Sil1. HspBP1 and Sil have characteristic Armadillo repeat folds (orange). All members of the BAG family in humans, Bag1-5, contain C-terminal Hsp70-binding BAG domains (red), but have otherwise divergent domain composition. Bag1 contains an Ubiquitin-like domain (Ubl, dark blue), which might associate with the regulatory particle of the 26S proteasome, and a NLS sequences (purple) for nuclear targeting. Bag2 contains a coiled-coil dimerization domain (CC, orange) (Page et al., 2012). Bag3 comprises multiple N-terminal sequence motifs including WW domains (WW, yellow), IPV sequence motifs (brown) and PXXP repeats (pink), which mediate interactions with proline-rich motifs, HspB8 and SH3 domains, respectively (Doong et al., 2000; Fuchs et al., 2010; Iwasaki et al., 2010; Ulbricht et al., 2013). Bag5 has four additional 3-helix bundle domains of unknown function (Arakawa et al., 2010). Bag6 is not shown because the original assignment as an NEF of Hsp70 was incorrect (Mock et al., 2015).

Molecular chaperones of the Hsp70 family use a nucleotide-dependent conformational cycle to support protein folding. Hsp70 proteins comprise an N-terminal nucleotide binding domain (NBD) and a substrate binding domain (SBD), which communicate by allosteric signals (reviewed in Mayer, 2013). The nucleotide is bound at the center of a two-lobed structure, commonly divided into subdomains IA, IB, IIA, and IIB. ATP binding favors a twist between the NBD lobes which allows extensive contacts with the SBD. In this conformation interactions with misfolded proteins are dynamic. In contrast, the ADP-bound state exhibits an extended structure with tight interactions between the SBD of Hsp70 and exposed hydrophobic peptide segments. ATP hydrolysis by Hsp70 thus induces stable substrate complex formation, and ATP re-binding triggers substrate release from Hsp70. Hsp70 has however high affinity for ADP and its intrinsic ATP hydrolase activity is low. Hence Hsp70 function critically depends on cochaperones, specifically J-domain proteins (JDP) and nucleotide exchange factors (NEFs), which accelerate ATP hydrolysis and ADP-ATP exchange, respectively. The great diversity of JDPs, especially in eukaryotes, suggests that Hsp70 is recruited for specific tasks by forming ternary complexes with substrates and these cochaperones. Eubacterial genomes encode only one NEF for Hsp70, GrpE. While in mitochondria and chloroplasts GrpE homologs are preserved, the cytosol and endoplasmic reticulum (ER) of eukaryotes contain three divergent families of NEFs: Hsp110/Grp170, HspBP1/Sil1 homologs and BAG-domain proteins (Höhfeld and Jentsch, 1997; Kabani et al., 2002; Steel et al., 2004; Dragovic et al., 2006; Raviol et al., 2006). The human genome encodes four Hsp110/Grp170 and two HspBP1/Sil1 homologs in addition to five BAG-domain proteins and two mitochondrial GrpE species (Figure 1). In addition, isoforms of NEFs resulting from alternative initiation sites and splicing exist. This NEF diversity may contribute to the appropriate allocation of Hsp70 folding capacity within the proteostasis network. Interestingly, metazoans, plants and some protists also harbor the additional cochaperone Hip (gene ST13), which antagonizes NEF function by stalling Hsp70 cycling and stabilizing Hsp70 complexes with specific substrates.

In this review we compare the structures and molecular mechanisms of different NEF families and discuss how these cochaperones contribute to protein remodeling, folding and quality control in the cell. For a more comprehensive overview, please see our earlier work (Bracher and Verghese, 2015).

## GrpE, the bacterial NEF

Protein folding by the eubacterial Hsp70 homolog DnaK in *Escherichia coli* depends on GrpE, which is encoded by an essential gene (Ang and Georgopoulos, 1989). Together with the JDP DnaJ, GrpE greatly accelerates ATP hydrolysis and thus conformational cycling of DnaK (Liberek et al., 1991; Laufen et al., 1999). However, it should be noted that *E. coli* also expresses two specialized Hsp70 isoforms, HscA and HscC, which do not depend on GrpE (Brehmer et al., 2001).

**Figure 2:**
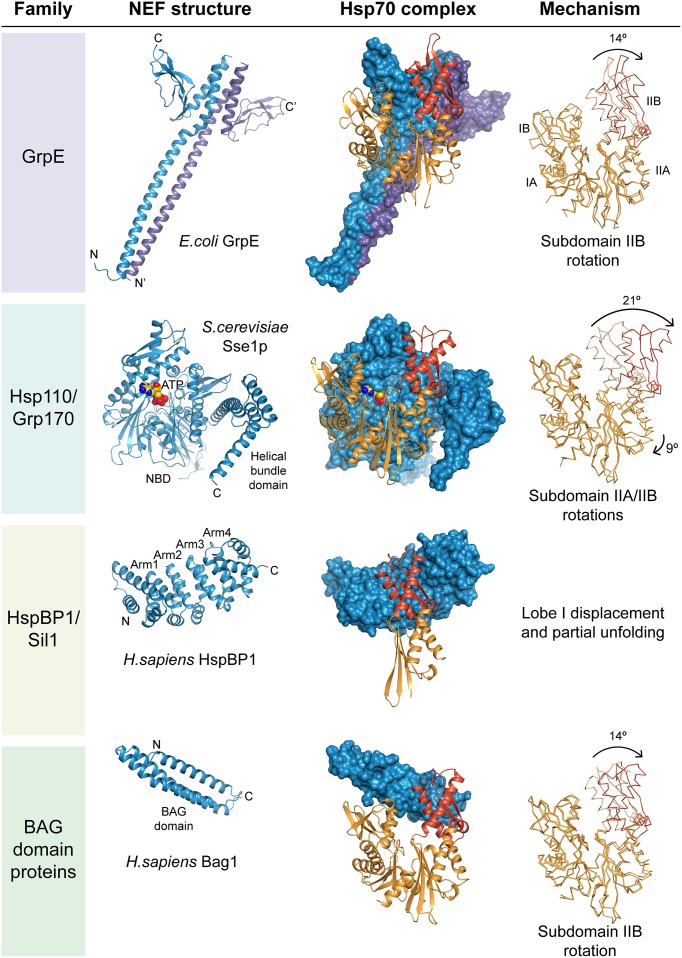
**Structure and mechanism of nucleotide exchange factors**. Exemplary structures of the four NEF families are shown together with respective Hsp70 complexes. The NEF is always shown in blue; the Hsp70 NBD in orange with subdomain IIB highlighted in red. On the right the structure of the NBD in the complex is superposed with the ADP-bound conformation, and the putative nucleotide exchange mechanism indicated. The drawings are based on the PDB coordinate sets 1DKG (GrpE•DnaK, Harrison et al., 1997), 2V7Y (DnaK•ADP, Chang et al., 2008), 3D2F (Sse1p•Hsp70, Polier et al., 2008), 1HPM (Hsc70•ADP, Wilbanks and Mckay, 1995), 1XQS (HspBP1•Hsp70-lobeII, Shomura et al., 2005) and 1HX1 (Bag1•Hsc70, Sondermann et al., 2001).

Structurally, GrpE is composed of an α-helical dimerization domain and a β-domain that mediates most of the interactions with DnaK (Harrison et al., 1997). The α-helical domains form a stalk-like, coiled-coil structure with a four-helix bundle at the C-terminal end; the β-domains protrude like wings from the helix bundle (Figure 2). In the complex with the NBD of DnaK, one β-domain inserts into the nucleotide binding cleft, forcing the nucleotide binding pocket open by rotation of subdomain IIB (Figure 2). This NBD conformation has greatly diminished affinity for nucleotide. Simulations of the highly conserved NBD suggest that subdomain IIB motion is facilitated by a flexible hinge connection (Ung et al., 2013). GrpE thus utilizes an in-built feature of Hsp70 for its function. In addition to the β-domain, parts of the stalk and flexible N-terminus of GrpE contribute to DnaK binding. The latter segment appears to compete with substrate binding to DnaK (Harrison et al., 1997).

GrpE function in *E. coli* appears to be regulated by temperature (Grimshaw et al., 2001). At the optimal growth temperature, GrpE is dimeric and active, enabling rapid DnaK cycling needed for folding newly synthesized proteins. Under thermal stress, the stalk of GrpE appears to unfold, which prevents the cochaperone from binding to DnaK•substrate complexes and thus limits futile cycling and ATP expenditure. During thermal stress, interactions with DnaK stabilize substrates against aggregation. The transition in GrpE is fully reversible and the DnaK-DnaJ-GrpE system begins to refold DnaK-stabilized substrates upon recovery.

## Eukaryotic GrpE homologs

Mitochondria and chloroplasts, the eukaryotic organelles of eubacterial origin preserve the bacterial Hsp70 system including GrpE homologs. The most conspicuous function of this system is the import of organellar proteins from the cytosol across membranes (see accompanying reviews). This is essential because most of the matrix/stroma proteins are encoded in the nucleus. Moreover, protein folding within these organelles also depends on GrpE function. The thermosensory function of GrpE appears to be preserved in eukaryotes (Moro and Muga, 2006; Willmund et al., 2007).

## Evolution of eukaryotic NEFs

In contrast to DnaK, authentic eukaryotic Hsp70 homologs have weakened contacts across the nucleotide binding cleft. Probably the function of the eukaryotic Hsp70 ancestor did not require NEFs. Dependence on NEFs apparently evolved only later, and thus modern GrpE and the mammalian NEF Bag1 function only with their cognate Hsp70 partners (Brehmer et al., 2001). In the presence of physiological phosphate concentrations, the ADP dissociation rates of eukaryotic Hsp70 diminish to the levels similar to DnaK in *E. coli*, and thus NEFs are required for efficient cycling *in vivo* (Gässler et al., 2001; Arakawa et al., 2011). The most ancient and universal type of eukaryotic NEF is Hsp110/Grp170, which shares its molecular architecture with canonical Hsp70 proteins (Figure 2). The other two NEF families, HspBP1/Sil1 homologs and BAG-domain proteins, have generic structural scaffolds, Armadillo repeats and three-helix bundles, respectively, which are also found in other functional contexts such as nuclear transport and vesicle fusion (Figure 2). Interestingly, despite their considerable structural diversity, all NEFs target subdomain IIB in the NBD of Hsp70. This suggests convergent evolution toward activating the molecular switch built into the Hsp70 NBD. It appears that the structurally homologous NEFs HspBP1 and Sil1p evolved independently, since their contact regions with the cognate Hsp70 homolog differ markedly (Shomura et al., 2005; Yan et al., 2011). Convergent evolution might also explain the perplexing diversity in BAG-domain architecture, which became only apparent when the individual structures were determined.

## Nucleotide exchange mechanism

All eukaryotic NEFs seem to capture open conformations of the NBD. GrpE literally drives a molecular wedge into the nucleotide binding cleft of DnaK, whereas the Hsp110 homolog in *Saccharomyces cerevisiae*, Sse1p, attaches to the flank of subdomain IIB while anchoring itself onto the remainder of the NBD (Polier et al., 2008). HspBP1/Sil1 homologs and BAG-domain proteins use their bulk mass to fix Hsp70 in an open conformation. HspBP1 binding even induces partial unfolding of the NBD. Because their shapes vary considerably, the NBD subdomains are displaced in different ways, as evidenced by cocrystal structures with Hsp70 NBDs (Figure 2). ATP but not ADP binding subsequently displaces NEFs from eukaryotic Hsp70 for a new round of substrate binding.

## Hsp110/Grp170

Hsp110 and its ER-lumenal homolog Grp170 share their domain architecture with canonical Hsp70 proteins, consisting of an N-terminal NBD, followed by a β-sandwich and an α-helix bundle domain, but have long insertions and C-terminal extensions compared to Hsp70 (Figure 1). Their sequence conservation is much lower than in canonical Hsp70. Crystal structures of the Hsp110 homolog Sse1p in complex with Hsp70 showed that the two NBDs face each other (Figure 2) (Polier et al., 2008; Schuermann et al., 2008). The NBD of Hsp70 is fixed in an open conformation by additional contacts with the α-helix bundle domain of Sse1p. These contacts are highly conserved in the Hsp110/Grp170 family, and presumably all members employ the same binding mode (Andreasson et al., 2010; Hale et al., 2010). To function, Sse1p requires ATP binding, which induces a compact conformation, but not ATP hydrolysis (Shaner et al., 2004; Raviol et al., 2006). Indeed, expression of ATP-binding competent, but ATPase-deficient Sse1p mutants rescues the lethal phenotype of the deletion of *SSE1*/*SSE2*. Because of low sequence conservation in the β-sandwich domain, it is unclear whether the substrate binding mode seen in DnaK·ADP is also employed by Hsp110/Grp170. Hsp110 homologs seem to prefer aromatic residues in target sequences, while canonical Hsp70 has a bias toward aliphatic hydrophobic and proline residues (Xu et al., 2012). Mammalian and yeast Hsp110 homologs are potent holdases for misfolded luciferase, preventing its aggregation until refolding with Hsp70/Hsp40 commences (Oh et al., 1997, 1999). Sse1p requires heat activation for this activity (Polier et al., 2010). Whether nucleotide-dependent cycling of Hsp110/Grp170 is required for holdase activity is controversial.

## HspBP1/Sil1

HspBP1 and Sil1 represent the cytosolic and ER-lumenal forms of a NEF family with Armadillo repeat architecture, respectively. Sil1 homologs can be identified in most eukaryotes; *Caenorhabditis elegans* apparently lacks a cytosolic HspBP1 homolog. The C-terminal 260-residue NEF domain of human HspBP1 consists of four Armadillo repeats capped at each end with α-helix pairs. The short N-terminal regions are highly divergent and their function is unknown. The structure of the human HspBP1 NEF domain in complex with a fragment of Hsp70 showed that the concave face of the Armadillo repeat structure wraps around subdomain IIB of the NBD (Figure 2) (Shomura et al., 2005). Comparison with other structures of the Hsp70 NBD suggests that the bulk of HspBP1 would clash with subdomain IB. Protease sensitivity and tryptophan fluorescence indicates that Hsp70 evades this through local unfolding in the complex. Surprisingly, the yeast ER paralog Sil1p employs different contacts and stops at mere opening of the nucleotide binding cleft (Yan et al., 2011). Interestingly, the interactions of metazoan Sil1 with the ER-Hsp70, BiP, seem more similar to HspBP1 than to the putative yeast ortholog Sil1p (Hale et al., 2010; Howes et al., 2012). Whether ATP binding pre-empts NBD unfolding under physiological conditions remains unknown.

## BAG domain proteins

The BAG domain-containing proteins represent the most divergent group among eukaryotic NEFs. Human Bag1 was the first eukaryotic NEF to be identified, after its initial characterization as the binding partner of anti-apoptotic protein Bcl-2 (Höhfeld and Jentsch, 1997). It contains a 3-helix bundle domain of 110 residues having NEF activity (Figure 2) (Sondermann et al., 2001). Subsequently four more homologs with putative Bag domains were identified in humans, Bag2-Bag5 (Takayama and Reed, 2001). All have distinct domain compositions (Figure 1). Structural analysis of the respective Bag domains revealed a surprising diversity of α-helix bundle architectures, but all share a conserved sequence signature that targets subdomain IIB of Hsp70. The Hsp70-binding BAG domains of Bag3, Bag4, and Bag5 form shorter 3-helix bundles than Bag1 (Briknarova et al., 2002; Brockmann et al., 2004; Arakawa et al., 2010). The “Brand New Bag” (BNB) domain of Bag2 has a dimeric four-helix bundle structure that can accommodate two Hsp70s (Xu et al., 2008). This could help target complexes of multiple Hsp70 molecules attached to one substrate molecule.

The diverse domains found together with the NEF domain in BAG proteins probably allow recruitment of Hsp70 for specific purposes. However, only the domain compositions of Bag1 and Bag3 appear conserved among metazoans. Bag1 homologs also seem to occur in plants (Kabbage and Dickman, 2008). Bag1 contains an Ubiquitin-like domain (Ubl) in addition to the NEF domain, suggesting a role in targeting substrates for proteasomal degradation. In murine Bag1, the Ubl domain also mediates interactions with EGF-like growth factor (Hung et al., 2014). Bag3 has multiple interaction motifs and connects Hsc70 with the small heat shock protein HspB8 and the dynein adaptor protein 14-3-3γ in targeting protein aggregates for degradation by autophagy via the microtubule network (Arndt et al., 2010; Fuchs et al., 2010; Xu et al., 2013). Bag2 forms stable ternary complexes with Hsc70 and the Hsp70-associated dimeric ubiquitin ligase CHIP, inhibiting proteasome targeting of inducible Hsp70 and substrate proteins (Arndt et al., 2005; Dai et al., 2005). The BNB domain has also been implicated in binding to substrate directly (Xu et al., 2008).

## Antagonism between Hip and NEF function

The dimeric multi-domain protein Hip antagonizes the function of Bag1 and probably other NEFs (Kanelakis et al., 2000). Hip slows the dissociation of ADP from Hsp70, thus stabilizing substrate protein association in presence of ATP (Höhfeld et al., 1995; Li et al., 2013). The crystal structure of the core complex of Hip with Hsp70·ADP revealed that the binding interfaces of Hip and NEFs overlap, resulting in mutually exclusive binding (Li et al., 2013). The binding affinity of NEFs — with the exception of Bag2 — is however ~100 times higher, and stable binding of Hip thus requires additional interactions. Simultaneous interactions with two Hsp70 molecules attached to a slow-folding substrate or an aggregate would boost affinity toward the Hip dimer through avidity. Hip might also directly recognize specific substrates such as the Hsp90-client protein glucocorticoid receptor via its DP domains (Nelson et al., 2004). Interaction of Hsp70-substrate complexes with Hip might thus enable slow cycling and limit futile energy expenditure by Hsp70 on ill-fated substrate proteins and downstream chaperone clients. Concomitantly, Hip binding might also prolong the time window for proteasomal targeting, consistent with increased disposal of mutant androgen receptor upon Hip overexpression (Wang et al., 2013).

## Involvement of NEFs in protein folding and import

In the model organism *S. cerevisiae*, Sse1p is by far the most abundant cytosolic NEF, followed by its inducible isoform Sse2p and the HspBP1 ortholog Fes1p. Together they constitute about 1/10 of the total concentration of cellular Hsp70 (Ghaemmaghami et al., 2003; Kulak et al., 2014). The Bag protein Snl1p is only present at low concentration and probably highly specialized (Verghese and Morano, 2012). Sse1p collaborates with ribosome-bound and cytosolic Hsp70 isoforms in folding and processing a large proportion of newly made proteins (Yam et al., 2005). Deletion of *SSE1* causes a growth defect, which is only partially rescued by Fes1p overexpression (Raviol et al., 2006). Overexpression of Sse1p impairs growth as well, suggesting that Sse1p competes with ATP binding to Hsp70 under these conditions (Liu et al., 1999). Well-balanced concentration ratios between Hsp70 and NEFs seem essential for proper protein folding. Sse1p, Sse2p, and Fes1p are upregulated together with Hsp70 under heat stress. Deletion of *FES1* causes a massive heat shock response in the absence of thermal stress (Gowda et al., 2013; Abrams et al., 2014), a folding defect of the reporter protein firefly luciferase (FLuc) (Shomura et al., 2005) and a thermosensitivity phenotype (Ahner et al., 2005). Besides their function in *de novo* protein folding, Sse1p and Fes1p also contribute in distributing substrates to downstream chaperones Hsp90 and TRiC and to clear misfolded species (Goeckeler et al., 2002; Mcclellan et al., 2005; Gowda et al., 2013).

The diversity of eukaryotic NEFs might enable adaptation of the Hsp70 cycling rate to the folding needs of specific substrate proteins. Consistent with this hypothesis, comparative *in vitro* studies with mammalian homologs showed that certain Hsp70/JDP/NEF combinations work much better in FLuc folding than others and some not at all (Tzankov et al., 2008; Rauch and Gestwicki, 2014). How specific combinations select suitable clients is unknown. NEF diversity might also allow differential distribution of substrates to downstream chaperones via adaptor proteins like HOP (Knapp et al., 2014). Functional redundancy of mammalian NEFs is, however, considerable. While knockout of the Hsp110 isoform Hsp105 causes no obvious defect (Nakamura et al., 2008), individual deletions of HspBP1 and Apg-2, another Hsp110 isoform, severely affect spermatogenesis (Held et al., 2011; Rogon et al., 2014). Only simultaneous knockout of Apg-1 and Apg-2 is lethal (Mohamed et al., 2014).

The ER-lumenal NEFs Sil1 and Grp170 contribute substantially to protein folding in the secretory pathway (Behnke et al., 2015). In addition they function together with BiP and the pore-associated JDP Sec63 in protein import, possibly by preventing substrate backsliding through BiP binding and release cycles (Zimmermann et al., 2011). Deletions of the respective NEF homologs in yeast, Sil1p and Lhs1p, activate the Unfolded Protein Response and cause import defects (Tyson and Stirling, 2000). Both Sil1 and Grp170 are upregulated under ER stress. In mice, knockout of Grp170 is lethal (Kitao et al., 2004). Mutations that inactivate Sil1 cause Marinesco-Sjögren syndrome in humans (Anttonen et al., 2005; Senderek et al., 2005) and the Woozy phenotype in mice (Zhao et al., 2005), respectively, conditions characterized by neurodegeneration and myopathy, likely because persistent ER stress induces apoptosis.

## Role in protein quality control

The folding of Cystic Fibrosis Transmembrane Conductance Regulator (CFTR) may serve as a paradigm for the role of NEFs in protein quality control involving proteasomal degradation. CFTR, a transmembrane protein, has inefficiently folding cytoplasmic domains, which may explain why a large proportion undergoes CHIP-mediated degradation before reaching the epithelial membrane (Meacham et al., 2001). Hsp105 appears to have a prominent role in CFTR folding at the ER and later at the epithelial membrane, employing its holdase activity (Saxena et al., 2012). Binding of HspBP1 to Hsc70 stimulates CFTR maturation (Alberti et al., 2004), whereas Bag1 collaborates with CHIP in CFTR degradation (Demand et al., 2001).

The alternative to proteasomal degradation is disposal of faulty proteins by the lysosome via autophagy. This route seems especially important for muscle structure maintenance, which requires the adaptor function of Bag3 (Arndt et al., 2010; Ulbricht et al., 2013). Bag3 deletion in mice results in severe myopathy (Homma et al., 2006). Similar phenotypes were reported for deletions of the probable *Drosophila melanogaster* and *C. elegans* orthologs, Starvin, and unc23, respectively (Arndt et al., 2010; Papsdorf et al., 2014). Bag3 mutations in humans are associated with autosomal dominant forms of myofibrillar myopathy and dilated cardiomyopathy (Selcen et al., 2009; Norton et al., 2011). Interestingly, Bag3 is the only stress-inducible BAG-domain protein (Franceschelli et al., 2008), and its increased abundance might tip the balance from proteasomal to lysosomal degradation (Gamerdinger et al., 2009).

Hsp110 proteins were found associated with aggregates of misfolding-prone proteins that cause neurodegenerative disease, including mutant SOD in Amyotrophic Lateral Sclerosis (Wang et al., 2009) and poly-Q androgen receptor in Spinal and Bulbar Muscular Atrophy (Ishihara et al., 2003). Hsp105-knockout mice accumulate hyper-phosphorylated tau similar to neurofibrillary tangles in Alzheimer's disease (Eroglu et al., 2010). Hsp110, together with Hsp70 and Hsp40, has been implicated in a metazoan disaggregase activity analogous to ClpB/Hsp104 in bacteria and fungi, which might resolubilize such aggregates for new folding attempts or proteasomal degradation (Shorter, 2011; Rampelt et al., 2012). Hsp110 thus may be considered the ultimate pro-folding NEF in eukaryotes, consistent with increased vulnerability of fast-growing cancer cells with a dominant-negative allele of this cochaperone (Dorard et al., 2011).

## Outlook

How the different components in the Hsp70 system intersect with other branches of the proteostasis network is only beginning to emerge. Different expression levels of competing NEFs and opposing factors like Hip may change the fate of specific substrate proteins in individual cell types. The intracellular distribution of proteostasis components might furthermore alter the dynamics of protein folding and degradation. Thus, information on cellular dose and distribution in healthy and diseased cells will be needed for an integrated picture of NEF roles in Hsp70-dependent cellular processes.

## Author contributions

Both authors contributed to drafting and critically revising the work.

### Conflict of interest statement

The authors declare that the research was conducted in the absence of any commercial or financial relationships that could be construed as a potential conflict of interest.
